# Cryo-EM structure of the bacterial divisome core complex and antibiotic target FtsWIQBL

**DOI:** 10.1038/s41564-023-01368-0

**Published:** 2023-05-01

**Authors:** Lisa Käshammer, Fusinita van den Ent, Magnus Jeffery, Nicolas L. Jean, Victoria L. Hale, Jan Löwe

**Affiliations:** MRC Laboratory of Molecular Biology, Francis Crick Avenue, Cambridge CB2 0QH, UK

**Keywords:** cell division, divisome, elongasome, cryo-EM, membrane proteins

## Abstract

In most bacteria, cell division relies on the synthesis of new cell wall material by the multiprotein divisome complex. Thus, at the core of the divisome are the transglycosylase FtsW, that synthesises peptidoglycan strands from its substrate Lipid II and the transpeptidase FtsI that crosslinks these strands to form a mesh, shaping and protecting the bacterial cell. The FtsQ-FtsB-FtsL trimeric complex interacts with the FtsWI complex and is involved in regulating its enzymatic activities, however, the structure of this pentameric complex is unknown. Here, we present the cryo-EM structure of the FtsWIQBL complex from *Pseudomonas aeruginosa* at 3.7 Å resolution. Our work reveals intricate structural details, including an extended coiled coil formed by FtsL and FtsB and the periplasmic interaction site between FtsL and FtsI. Our structure explains the consequences of previously reported mutations and we postulate a possible activation mechanism involving a large conformational change in the periplasmic domain. Since FtsWIQBL is central to the divisome, our structure is foundational for the design of future experiments elucidating the precise mechanism of bacterial cell division, an important antibiotic target.

## Main

Cell division, or cytokinesis is a fundamental process of life and, in most bacteria, is driven by peptidoglycan synthesis at the septum^1^. It is catalysed by the divisome, a multi-protein complex with more than 20 components that spans the cell envelope in bacteria harbouring a cell wall^2^.

**Fig. 1:**
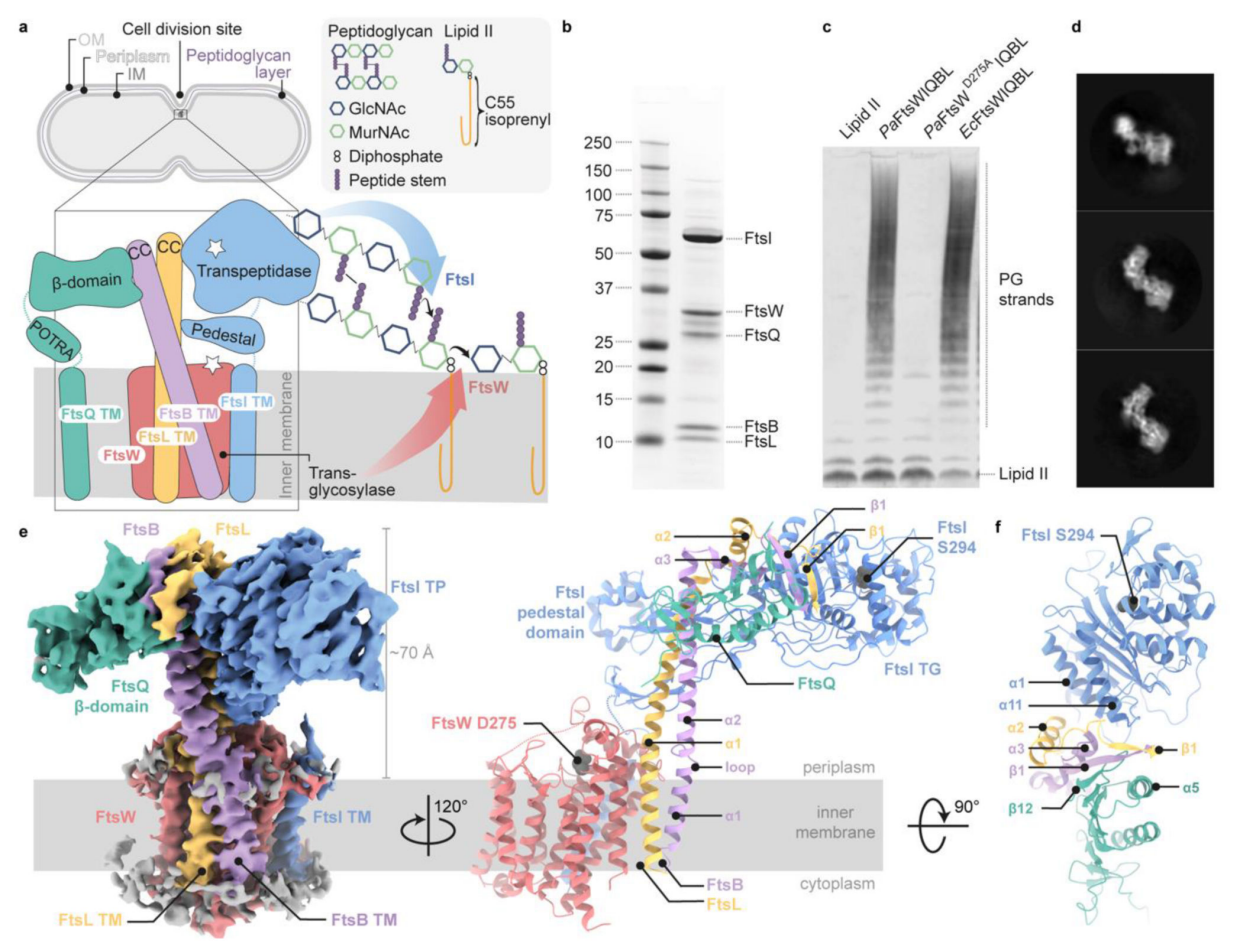
Biochemical and structural characterisation of the core divisome complex FtsWIQBL from *P. aeruginosa*. **a)** Septal peptidoglycan synthesis by FtsWIQBL during Gram-negative bacterial cell division. The transglycosylase FtsW (red), and transpeptidase FtsI (blue) bind the non-enzymatic subcomplex FtsQBL (green, violet and yellow, respectively). The complex contains 14 transmembrane helices – ten from FtsW and one each from FtsIQBL. The transglycosylase FtsW catalyses the polymerisation of GlcNAc-MurNAc disaccharides from Lipid II. The transpeptidase FtsI crosslinks the peptides from the nascent chain to adjacent peptides in the peptidoglycan layer between residues three and four. OM: outer membrane, IM: inner membrane, GlcNAc: N-acetylglucosamine, MurNAc: N-acetylmuramic acid, CC: coiled coil, TM: transmembrane. **b)** SDS-PAGE of the co-purified *Pa*FtsWIQBL complex after size-exclusion chromatography. **c)** Western blot showing glycan strand ladders synthesised by divisome core complexes from Lipid II, demonstrating transglycosylase activity. The negative control does not contain any FtsWIQBL (lane 1). WT *P. aeruginosa* and *E. coli* FtsWIQBL complexes (lanes 2 and 4) are active transglycosylases, while the *P. aeruginosa* putative active site mutant FtsW^D275A^IQBL (lane 3) is inactive. **d)** Three representative 2D classes from our *Pa*FtsWIQBL cryo-EM data. **e)** Left panel: side-view of the *Pa*FtsWIQBL cryo-EM density at an overall resolution of 3.7 Å. Protein colours are the same as those in a). Residual density from the detergent micelle is visible around the transmembrane domain and shown in grey. Right panel: model of *Pa*FtsWIQBL, rotated by 120° with respect to the density on the left-hand side. The putative FtsW active site residue D275 is indicated, as is the FtsI active site residue S294. FtsW loop 219-233 and FtsI loop 45-50 are shown as dotted lines as they were too flexible to build. FtsQ™ and FtsQ^β^ were not resolved and are not shown. **f)** Top view of the periplasmic domain, showing interactions between FtsI, FtsL, FtsB and FtsQ.

Central to the divisome is the peptidoglycan-synthesising protein complex FtsWI, with the transglycosylase (TG) FtsW polymerising glycan strands from its substrate Lipid II^3,4^, and the transpeptidase (TP) FtsI crosslinking peptide stems, thus forming a covalent mesh between glycan strands^5,6^ (Fig. 1a). Septal peptidoglycan synthesis occurs after activation of the divisome glycosyltransferase-transpeptidase pair FtsWI^3^, in particular through an interaction with the heterotrimer FtsQBL, as has been shown *in vitro*^7^.

Here, we present the cryo-EM structure of the catalytic divisome core complex FtsWIQBL from *Pseudomonas aeruginosa* at 3.7 Å resolution. The structure reveals details of the periplasmic interfaces within FtsWIQBL, including the positioning of FtsI by the coiled coil of FtsBL, as well as a transmembrane domain containing FtsWIBL but not FtsQ. With our structure we are able to provide explanations of a multitude of known mutations that interfere with divisome activation and regulation. Finally, we suggest the existence of a large conformational switch between presumably inactive and active states of the FtsWI core enzymes.

Our work is foundational for further structural, biochemical and genetic studies elucidating the molecular mechanisms of bacterial cell division. Since the divisome peptidoglycan synthase is essential for cell division in most bacteria, and is absent in eukaryotic cells entirely, it is a key target of important existing antibiotics and new antibiotic development^8^. Our structure will help to accelerate these efforts.

### Cryo-EM structure of the core divisome complex FtsWIQBL

**Extended Data Fig. 1:**
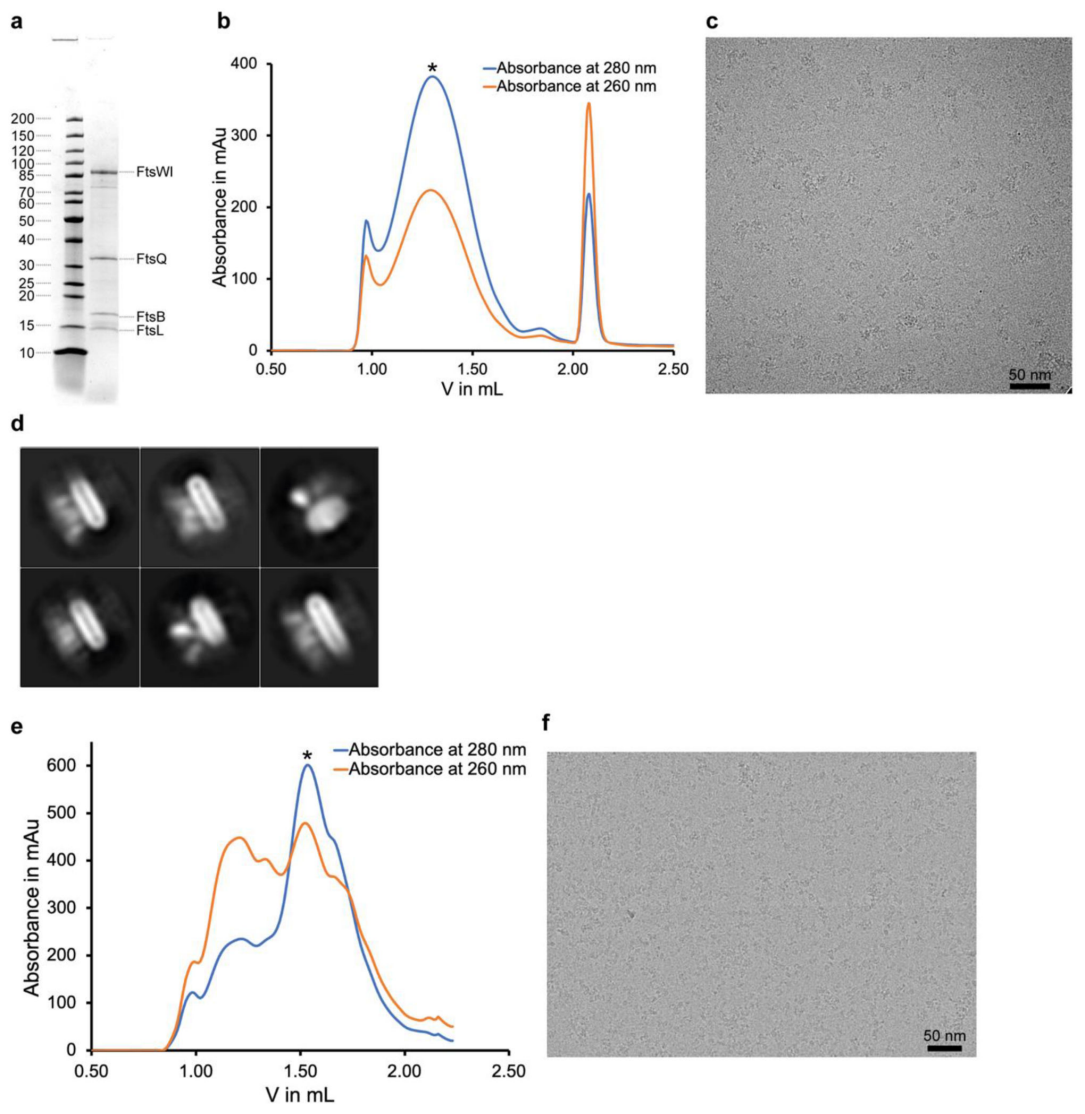
Protein purification and grid preparation of FtsWIQBL from *E. coli* (*Ec*) and *P. aeruginosa* (*Pa*). **a)** SDS-PAGE gel of the co-expressed and purified *Ec*FtsWIQBL divisome core complex after size-exclusion chromatography in GDN. FtsW and FtsI are joined by a short linker in this construct. **b)** Size-exclusion chromatogram of *Ec*FtsWIQBL reconstituted in nanodiscs. The measured absorbances at 260 nm and 280 nm are shown in orange and blue, respectively. The peak fraction of the size exclusion run, indicated with an asterisk, was used for grid preparation. **c)** Representative micrograph of *Ec*FtsWIQBL in nanodiscs. **d)** Representative 2D Classes of *Ec*FtsWIQBL in nanodiscs. **e)** Size-exclusion chromatogram of *Pa*FtsWIQBL. The measured absorbances at 260 nm and 280 nm are shown in orange and blue, respectively. The peak fraction of the size exclusion run, indicated with an asterisk, was used for grid preparation. **f)** Representative micrograph of *Pa*FtsWIQBL used for the final reconstruction.

**Extended Data Fig. 2:**
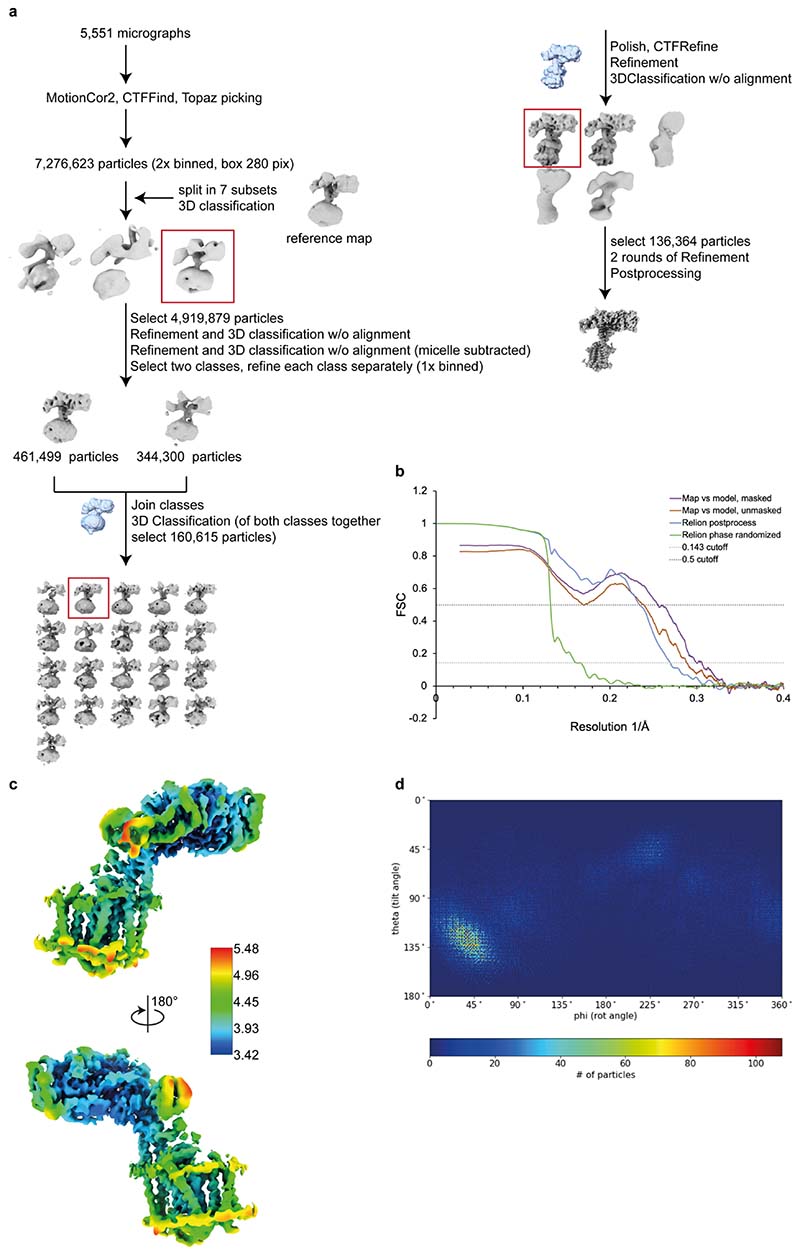
Details of the *Pa*FtsWIQBL cryo-EM processing. **a)** Cryo-EM image processing scheme for *Pa*FtsWIQBL. **b)** Fourier Shell Correlation (FSC) curves for the *Pa*FtsWIQBL cryo-EM maps and structures. **c)** The local resolution of the final structure of *Pa*FtsWIQBL was calculated with RELION’s own implementation and depicted in ChimeraX. **d)** The angular distribution of the particles making up the final structure of *Pa*FtsWIQBL.

**Extended Data Fig. 3:**
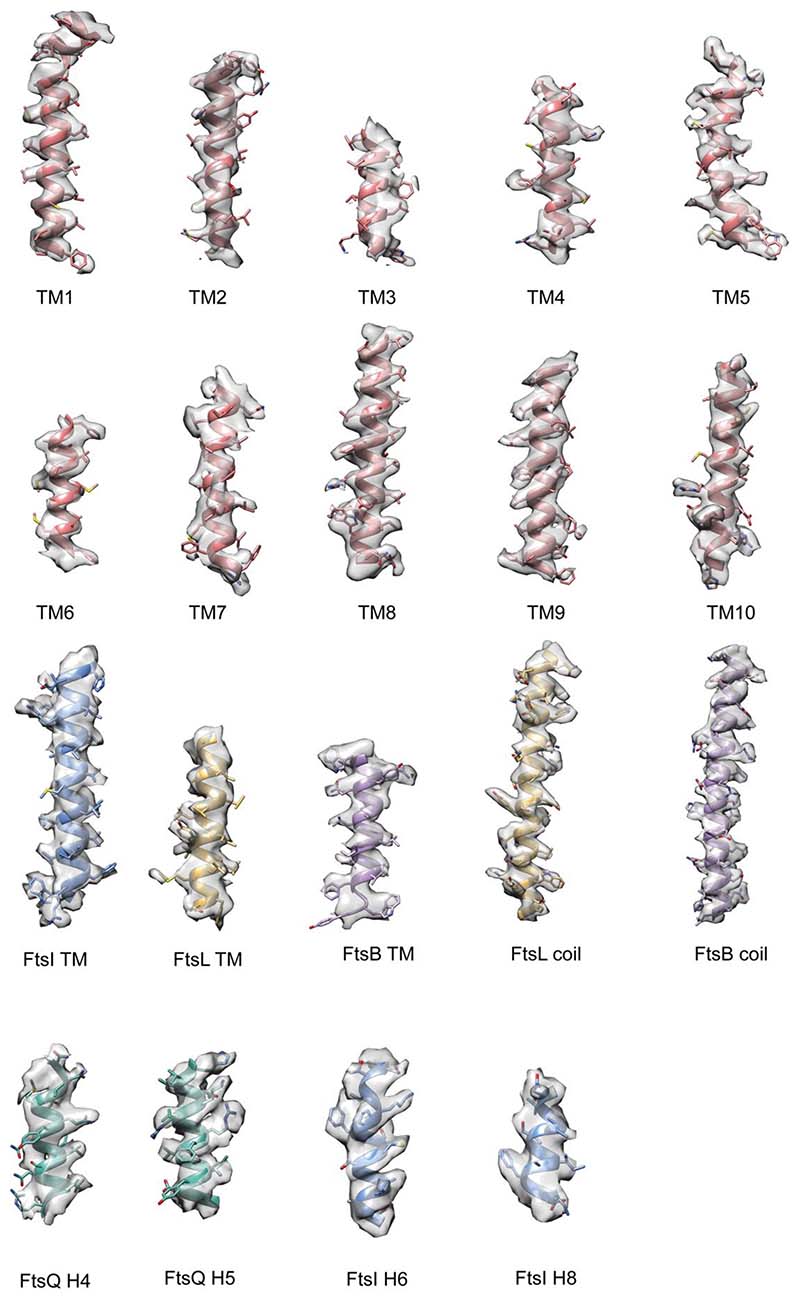
Details of the cryo-EM density around helices of *Pa*FtsWIQBL. All TM helices visible in the structure are shown, as well as the FtsL^α1^ and FtsB α2 coils, two helices from FtsQ^β^ and two helices from the FtsI^TP^ domain.

To solve the structure of the core divisome complex, we initially purified the *Escherichia coli* *Ec*FtsWIQBL complex expressed in insect cells (Extended Data Fig. 1a). Several approaches, including detergents, amphipoles and reconstitution in nanodiscs were employed in attempts to obtain a high resolution *Ec*FtsWIQBL structure. The best cryo-EM results of the *Ec*FtsWIQBL sample were achieved in nanodiscs (Extended Data Fig. 1b-d), however, due to variability in the obtained nanodisc size and composition, as well as strong alignment on the nanodisc during processing we were unable to solve the *Ec*FtsWIQBL structure. We therefore switched to the *Pseudomonas aeruginosa* *Pa*FtsWIQBL complex expressed in *E. coli* (Fig. 1b and Extended Data Fig. 1e). Both *Ec*FtsWIQBL and *Pa*FtsWIQBL possess comparable transglycosylase activity, while the putative active site mutant *Pa*FtsW^D275A^IQBL is inactive (Fig. 1c). This is in accordance with previous data where the *Pa*FtsW^D275A^IQBL mutant displayed reduced transglycosylase activity *in vitro* and caused filamentation in *P. aeruginosa* cells when overexpressed^*3*^. Having confirmed that our purified *Pa*FtsWIQBL complex produced peptidoglycan strands, we proceeded with single-particle averaging cryo-EM and determined the structure of *Pa*FtsWIQBL to a final overall resolution of 3.7 Å (Fig. 1d, e, and Extended Data Fig. 1f, 2, 3, Supplementary Table S1).

### General architecture of the FtsWIQBL complex

**Extended Data Fig. 4:**
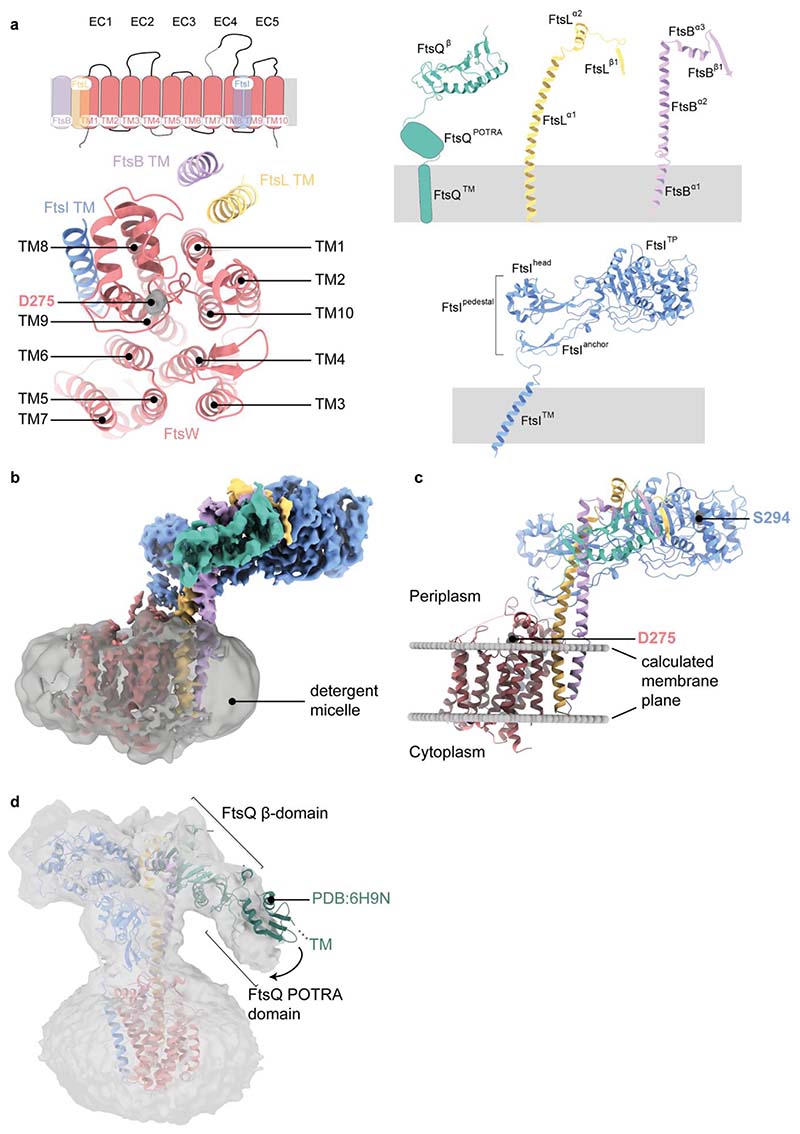
Architecture of the *Pa*FtsWIQBL complex. **a)** Upper left panel: schematic of the transmembrane helices of FtsW, FtsI, FtsL and FtsB. Two extracellular loops of FtsW that could not be build due to missing density and the N- and C-terminal tails of FtsW are indicated by doted lines. Lower left panel: top view of the transmembrane domain, with FtsW transmembrane helices consecutively numbered based on the sequence (identical to numbering of helices in a previous RodA structure^23^). FtsW’s putative active site residue D275 is indicated. Right panel: Labelling of the different domains in FtsQ, FtsL, FtsB and FtsI that was used throughout the paper. **b)** Cryo-EM density showing *Pa*FtsWIQBL within the Lauryl Maltose Neopentyl Glycol (LMNG) detergent micelle, which was subtracted during the later processing stages. **c)** Prediction of the position and orientation of the divisome core complex transmembrane segments in the lipid bilayer using the Orientations of Proteins in Membranes webserver^9^. The membrane plane is indicated with two grey discs and the active sites of FtsW and FtsI are labelled. **d)** A low-resolution structure obtained after fewer 3D classifications shows additional density for FtsQ^POTRA^ at low contour levels and indicates that the transmembrane segment of FtsQ is most likely not part of the micelle that contains the other TM segments. Alignment of a previous FtsB:FtsQ crystal structure (PDB: 6H9N) on FtsQ^β^ shows that FtsQ^β^ and FtsQ^POTRA^ adopt different conformations relative to each other.

All five proteins were resolved in the final cryo-EM reconstruction (Fig. 1e), with FtsQ being partially disordered. The density for the membrane domain of *Pa*FtsWIQBL reveals 13 transmembrane (TM) helices, including ten helices from FtsW, plus one each from FtsI, FtsB and FtsL (Extended Data Fig. 4a). Density for the FtsQ transmembrane helix (FtsQ™) was not observed (Extended Data Fig. 4b). The detergent micelle density was subtracted from the final reconstruction and the position of the complex in the membrane was approximated using the Orientations of Proteins in Membranes (OMP) webserver^9^ (Fig. 1e and Extended Data Fig. 4b, c).

The periplasmic domains of the *Pa*FtsWIQBL complex extend about 70 Å away from the membrane in a “Y”-shape, with the FtsI transpeptidase domain (FtsI^TP^) and the FtsQ β-domain (FtsQ^β^) located on opposite arms of the Y, and FtsBL connecting them (Fig. 1e, f). Interestingly, only FtsQ^β^ is well-resolved, while density for the FtsQ polypeptide-transport-associated domain (FtsQ^POTRA^) is only visible in low-resolution maps at high contour level, and density for FtsQ™ is completely absent (Extended Data Fig. 4d). FtsQ^POTRA^ adopts a slightly different orientation relative to FtsQ^β^ compared to previously determined X-ray structures^10–12^ (Extended Data Fig. 4d). Taken together, this shows that FtsQ is tethered to FtsWILB via its FtsQ^β^-FtsB interaction, while FtsQ^POTRA^ and FtsQ™ are flexibly attached in the current complex (Fig. 1f). As FtsQ™ is not visible, we assume it is not in the micelle that contains the other TM segments but might be surrounded by detergent molecules separately. While it has been previously reported that FtsB dimerises and could thus facilitate the dimerisation of core divisome components^13,14^, we find no evidence for higher oligomeric species in our cryo-EM data. We cannot exclude dimerisation of FtsB on its own, but in our current structure dimerisation *via* FtsB would be hindered by the presence of FtsI and/or FtsQ.

**Extended Data Fig. 5:**
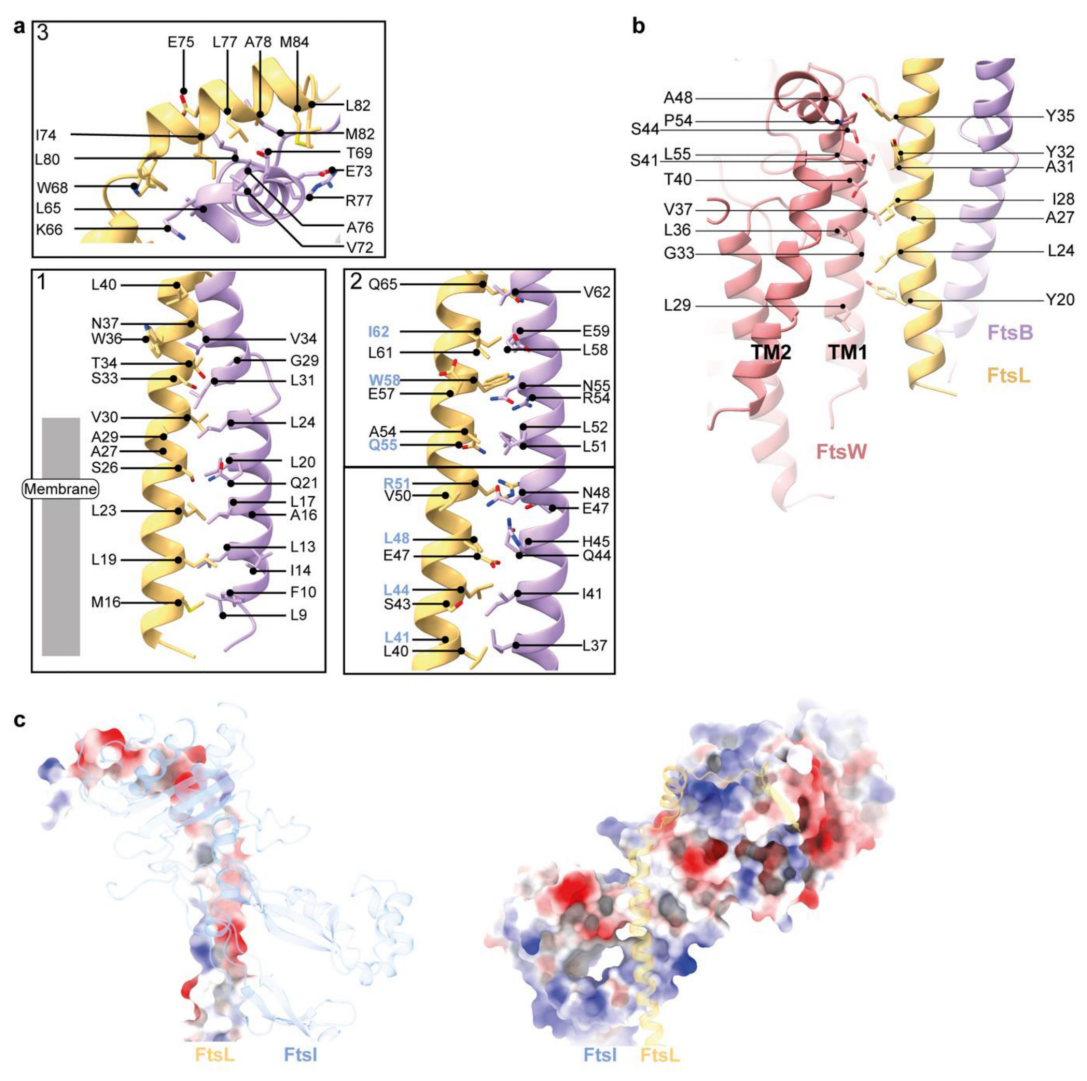
Detailed analysis of the interactions between FtsB and FtsL, FtsW and FtsL and FtsI and FtsL. **a)** Interaction sites between FtsB and FtsL, as also indicated in Figure 2a. Residues of FtsL that also interact with FtsI are highlighted in blue. **b)** Analysis of the interaction sites between FtsL and FtsW in their transmembrane region. The coiled coil conformation of FtsL means that the interaction surface is not as extended as it would be if it were straighter and not in a coiled coil. **c)** Electrostatic analysis of the interactions between FtsI and FtsL shows that the interaction site in the coiled coil area is mainly hydrophobic/neutral.

**Fig. 2:**
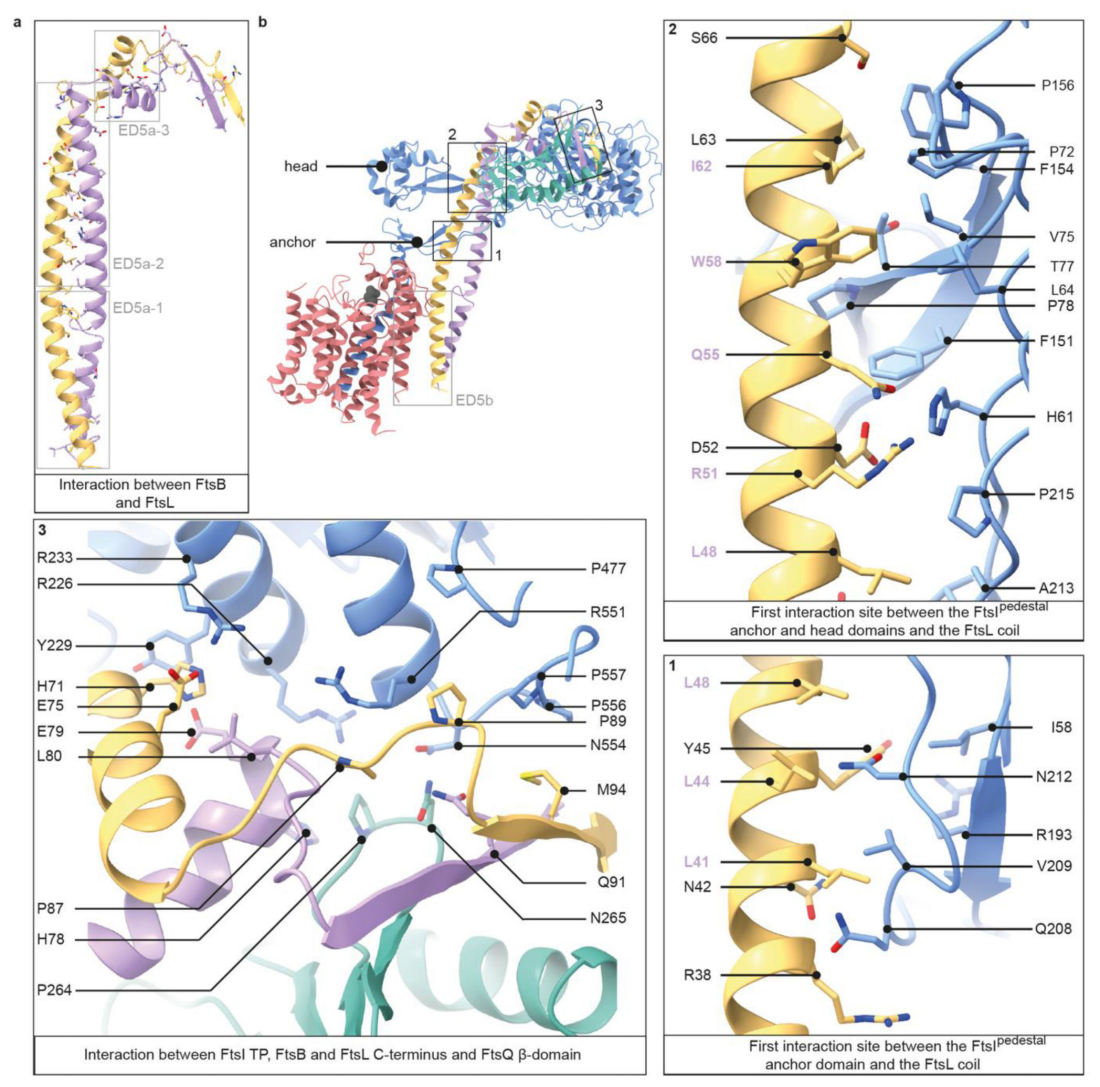
Interactions between FtsI, FtsQ, FtsB, and FtsL. **a)** FtsB and FtsL adopt a similar fold and interact with each other over their whole length. Grey boxes indicate regions that are further discussed in Extended Data Fig. 5a (ED5a). **b)** Key interactions within the FtsWIQBL periplasmic domain are highlighted in boxes 1 to 3. The grey box indicates a region that is further discussed in Extended Data Fig. 5b (ED5b). Panels 1-2: the interactions between FtsI^pe^destal and the FtsL coiled coil are shown in two panels. Most residues in the interface between FtsI and FtsL in this region are hydrophobic or neutral. Residues in FtsL that also face FtsB are highlighted in violet. Panel 3: the interaction between FtsI, FtsL, FtsB and FtsQ as seen from the top of the periplasmic domain.

FtsL and FtsB have similar folds, each consisting of a long α-helical coiled coil segment, followed by a short α-helix and a β-strand. Interestingly, the FtsB α-helical coiled coil is interrupted by a small, conserved loop just above FtsB™ that might aid with sterically maintaining the correct insertion depth in the membrane (Fig. 1e, 2a). The observed interruption of the FtsB coiled coil has been postulated previously using computational models^14,15^. FtsB and FtsL interact with each other over their entire lengths through mainly hydrophobic interactions, e.g. between FtsL^α1^ and FtsB^α2^ (Fig. 2a and Extended Data Fig. 5a). Thus, our structure clarifies the FtsL-FtsB interaction and confirms previous reports that suggested a coiled-coil interaction between FtsB and FtsL^16,17^.

The transglycosylase FtsW and transpeptidase FtsI share two interfaces. The first interface is in the membrane, where FtsI™ interacts with TM8 and TM9 of FtsW (Extended Data Fig. 4a) – an interaction that closely resembles that of the previously reported RodA-PBP2 elongasome complex from *Thermus thermophilus*^18^. The second interaction site is located between the extracellular loop 4 of FtsW (FtsW^EC4^) and the linker between FtsI™ and FtsI^pedestal^. Due to the flexibility of FtsI in this region, not all contacts could be determined unambiguously.

It has been previously reported that the cytoplasmic tail of *E. coli* FtsL is required for the recruitment of FtsW^19^. In the structure presented here, the FtsL cytoplasmic tail could not be traced unambiguously. This could either point towards a transient interaction during recruitment or species-specific differences in the recruitment due to the size of the cytoplasmic tail (11 residues in *P. aeruginosa* vs. 34 residues in *E. coli*). However, we clearly observe FtsL-FtsW interactions in the periplasm (FtsW^EC1^ and FtsL^α1^), and within the membrane through FtsW^TM1^ and the upper three turns of FtsL™. In the latter, the lower part of FtsL™ twists away from FtsW, due to its gyrating coiled-coil interaction with FtsB (Extended Data Fig. 5b).

### FtsIQBL interactions in the periplasm

FtsL and FtsI form an extensive interface in the periplasm, with a total buried surface area of 1035 Å^2^. The FtsL-FtsI interaction is facilitated by two sites: FtsL^α1^-FtsI^pedestal^, involving anchor and head subdomain residues in FtsI^pedestal^, and FtsL^α2,β1^-FtsI^TP^ (Fig. 2b and Extended Data Fig. 5c). Moving along the FtsL coil, the first mainly hydrophobic and neutral interactions occur between FtsL^α1^ (residues L41-L48) and the anchor subdomain of FtsI^pedestal^ (residues I58, R193, Q208-215, Fig. 2b, panel 1). FtsI^pedestal^ slightly wraps around FtsL^α1^, forming a hydrophilic interaction site (FtsI H61 with FtsL R51, D52 and Q55, Fig. 2b panel 2). The final, and mainly hydrophobic, interaction site on FtsL^α1^ involves residues A56-S66 and residues located mainly in the FtsI^pedestal^ head subdomain (L64, P72-P78, F151-P156, Fig. 2b panel 2). Importantly, no direct interaction was observed between the FtsB coiled coil and FtsI (Fig. 2b and Extended Data Fig. 5a).

**Extended Data Fig. 6:**
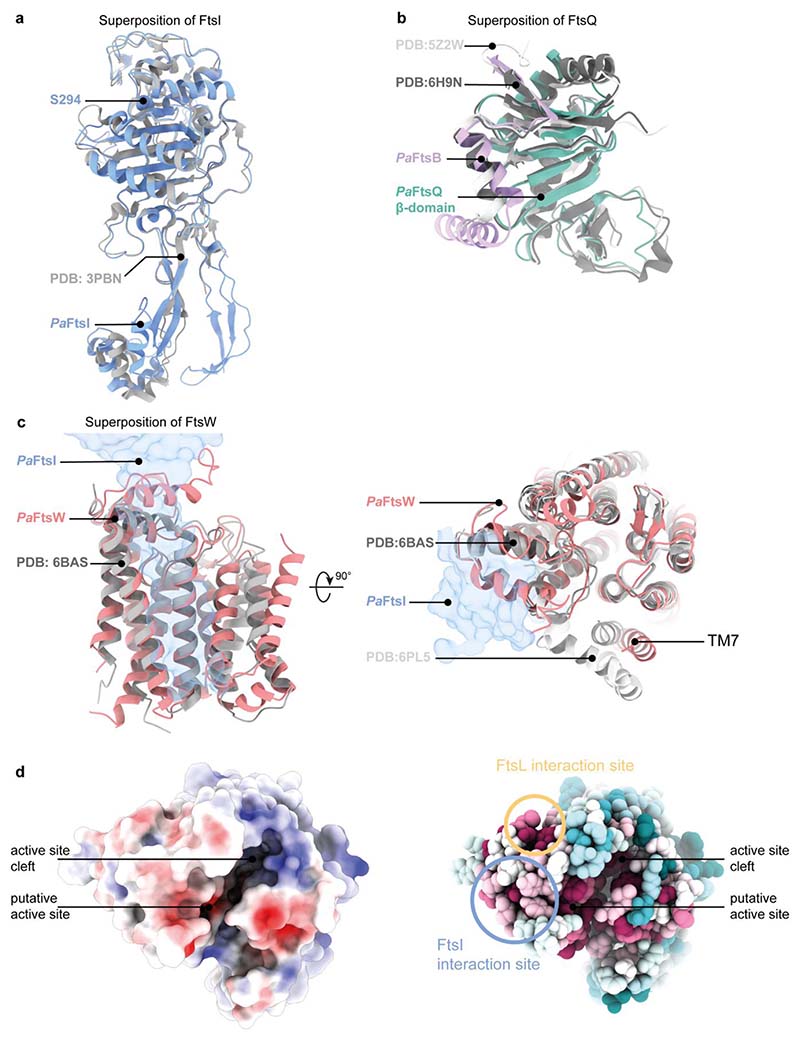
Comparison of *Pa*FtsWIQBL cryo-EM structure with previous crystal structures of FtsI, FtsQ and FtsW/RodA. **a)** Superposition of FtsI determined by X-ray crystallography (PDB: 3PBN, grey) with the FtsI part of *Pa*FtsWIQBL cryo-EM structure. The TP active site residue S294 is indicated (RMSD of 0.708 Å across 372 pruned atom pairs). **b)** Superposition of FtsQB determined by X-ray crystallography (PDB: 6H9N in dark grey, PDB: 5Z2W in light grey) with the same area in the cryo-EM structure determined here (For alignment of FtsQ: RMSD (FtsQ-6H9N) of 1.186 Å across 86 pruned atom pairs, RMSD (FtsQ-5Z2W) of 1.118 Å across 95 pruned atom pairs). **c)** Superposition of RodA determined by X-ray crystallography (PDB: 6BAS in dark grey (left and right), PDB: 6PL5 in light gray (right)) and FtsW in the cryo-EM structure. The position of FtsI is indicated as a transparent blue outline. Apart from transmembrane helix 7, the structures align very well (RMSD (FtsW-6PL5) of 1.188 Å across 202 pruned atom pairs; RMSD (FtsW-6BAS) of 1.126 Å across 206 pruned atom pairs). **d)** Electrostatic surface representation of *Pa*FtsW viewed from the periplasmic side. A deep cleft is visible that contains the putative active site residue D275. The same representation showing sequence conservation of FtsW mapped onto the surface representation shows that this cleft is highly conserved. Additionally, interaction sites with FtsI and FtsL are indicated; these also show above average levels of sequence conservation.

The second FtsL–FtsI interaction site is located on top of the periplasmic domain (Fig. 2b panel 3): FtsL^α2,β1^-FtsI^TP^. H71 of FtsL^α2^ stacks against Y229 of FtsI^TP^ and is flanked by additional residues in FtsB (E79, L80), FtsI (R233) and FtsL (E75). An additional hydrophobic interface site is formed by several proline residues in both FtsL and FtsI^TP^ [P556, P557 (FtsI) – M94 (FtsL); P557, G478, P477 (FtsI) – P89 (FtsL); R551 (FtsI) - P87 (FtsL)]. Interestingly, FtsB^α3^ and FtsB^β1^ frame a loop in FtsQ between β-strands 11 and 12, forming the only interaction site between FtsI, FtsB and FtsQ [N554 (FtsI) – N265 (FtsQ) – Q91 (FtsB); R226 (FtsI) - P264 (FtsQ); R226 (FtsI) – H78 (FtsB, backbone), R551 (FtsI) – L80 (FtsB, backbone)]. FtsI adopts a structure very similar to previously reported crystal structures^20^, with only minor changes in the FtsI^pedestal^ domain, indicating that FtsL binding does not cause large rearrangements in FtsI^TP^ (Extended Data Fig. 6a).

Only a few FtsL-FtsQ contacts are present, however FtsL completes an extended β-sheet formed between FtsQ^β^ strands β5 to β12 and FtsB^β1^, by contributing its last β-strand (Fig. 1f, Fig. 2b panel 3). The FtsB-FtsQ interaction recapitulates that of previously determined crystal structures, where only small parts of FtsB^11,12^ were resolved (Extended Data Fig. 6b). In addition, the cryo-EM structure shows an interaction between FtsB^α2^ (starting from E53) and FtsQ^β^ loops (R183, S212-R214, R231). Interruption of the FtsB-Q interface with inhibitors based on the minimal interface could be expected to also disrupt the interface in the context of the divisome core complex^21^.

### Comparison with RodA-PBP2 structures and AF2 predictions

Cell elongation in rod-shaped bacteria is facilitated by the elongasome that, like the divisome, polymerises and crosslinks PG, but is positioned throughout the cell envelope by moving MreB filaments^22^. RodA, the elongasome’s transglycosylase is related to FtsW and has previously been structurally characterised using X-ray crystallography both on its own and as a RodA-PBP2 complex^18,23^ (the latter being homologous to FtsWI). The structures of *Pa*FtsW determined here and *Tt*RodA are very similar, with the exception of TM7, which appears to be somewhat flexible in the cryo-EM structure, straighter with respect to that of *Tt*RodA and closer to TM5 than in *Tt*RodA-PBP2 (Extended Data Fig. 6c). It has been postulated that the movement of TM7 could open a cavity for the binding of the lipid tail of Lipid II to RodA^18^ and the location of TM7 in *Pa*FtsWIQBL creates such a cavity. The putative catalytic residue D275A is located in a deep, highly conserved cleft, as shown in Extended Data Fig. 6d, that we suggest might harbour the sugar moieties of Lipid II during the transglycosylase reaction.

**Extended Data Fig. 7:**
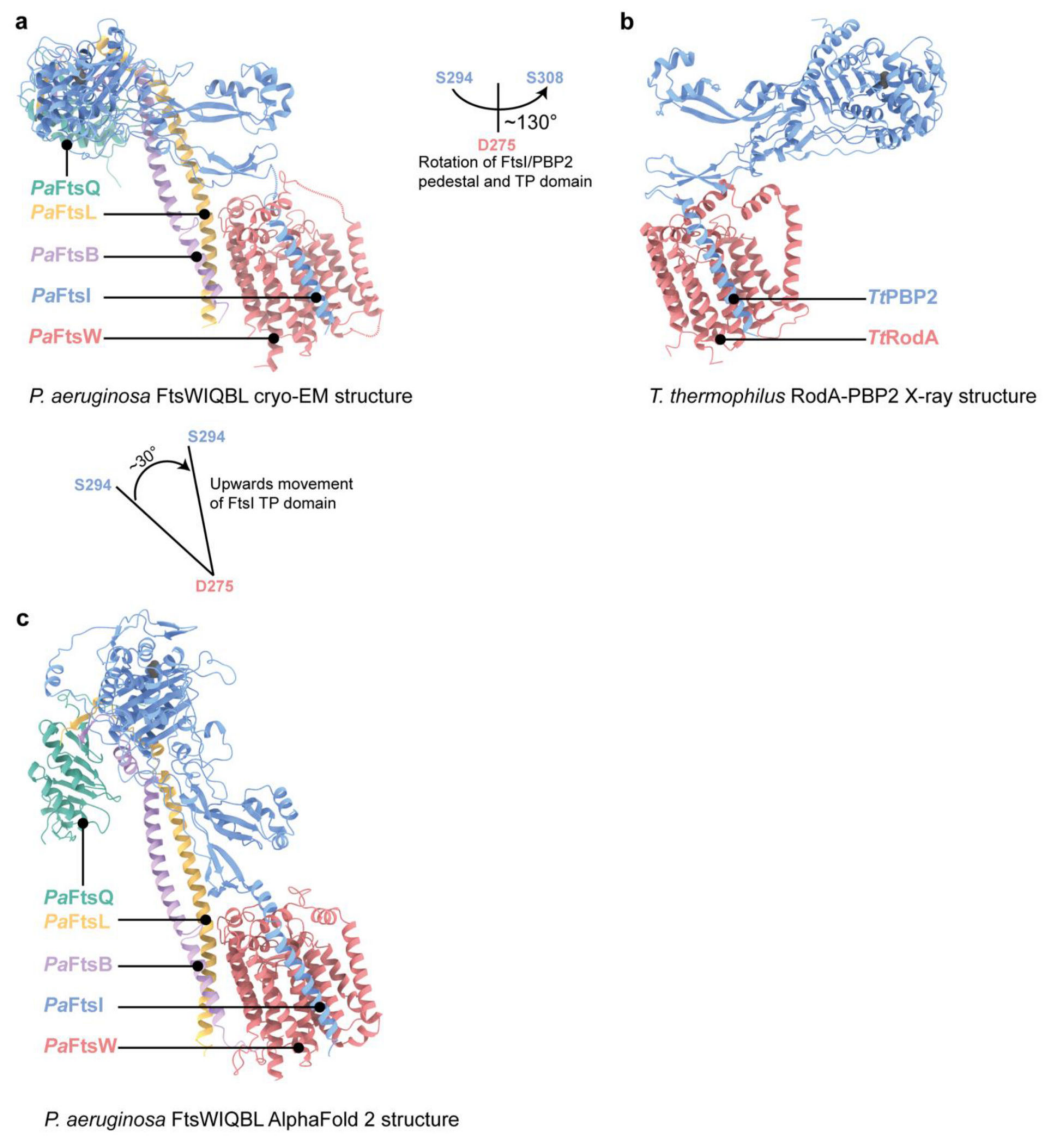
Comparison of the *Pa*FtsWIQBL cryo-EM structure with RodA-PBP2 structure and AlphaFold2 structure prediction. Comparison of the cryo-EM structure *Pa*FtsWIQBL **(a),** the *Thermus thermophilus* RodA-PBP2 crystal structure (PDB: 6PL5, **b)** and the AlphaFold2 prediction of *Pa*FtsWIQBL **(c)**. All three structures were aligned on FtsW/RodA. The FtsQ^POTRA^ and FtsQ™ of the AlphaFold2 model were removed for clarity. The FtsI/PBP2 periplasmic domains show a large 130° lateral rotation between the *P. aeruginosa* FtsWI and *T. thermophilus* RodA-PBP2 models (a-b). The rotation was measured around an axis perpendicular to the membrane plane and intersecting the FtsW active site. The distance between both active sites in FtsI (S294) and PBP2 (S308) is 125 Å. A 30° vertical rotation of the periplasmic FtsI domains is visible between the cryo-EM and AlphaFold2 models of *Pa*FtsWIQBL (a-c). The angle was measured between the FtsW active site (D275) and the FtsI active sites (S294). The distance between the FtsI active sites in the cryo-EM and AlphaFold2 models is 46 Å.

The most striking observation when comparing *Pa*FtsWIQBL with *Tt*RodA-Pbp2 is the difference in the relative orientations of the TP with respect to the TG domain, despite the fact that the structures of the single proteins superimpose well on their own. Alignment of both complexes on the FtsW/RodA subunits places *Pa*FtsI^TP^ and *Tt*PBP2^TP^ almost opposite to each other, requiring a ~130° rotation of *Pa*FtsI^TP^/*Tt*PBP2^TP^ for their interconversion (Extended Data Fig. 7a, b). The reason for this large difference is unclear, but is possibly caused by the presence of FtsQBL in our divisome structure and the absence of binding partners such as MreCD in the elongasome structure. Alternatively, the differences could be intrinsic to the elongasome and divisome complexes or reflect different, distinct states in the regulatory/catalytic cycle of the enzyme complexes.

**Fig. 3:**
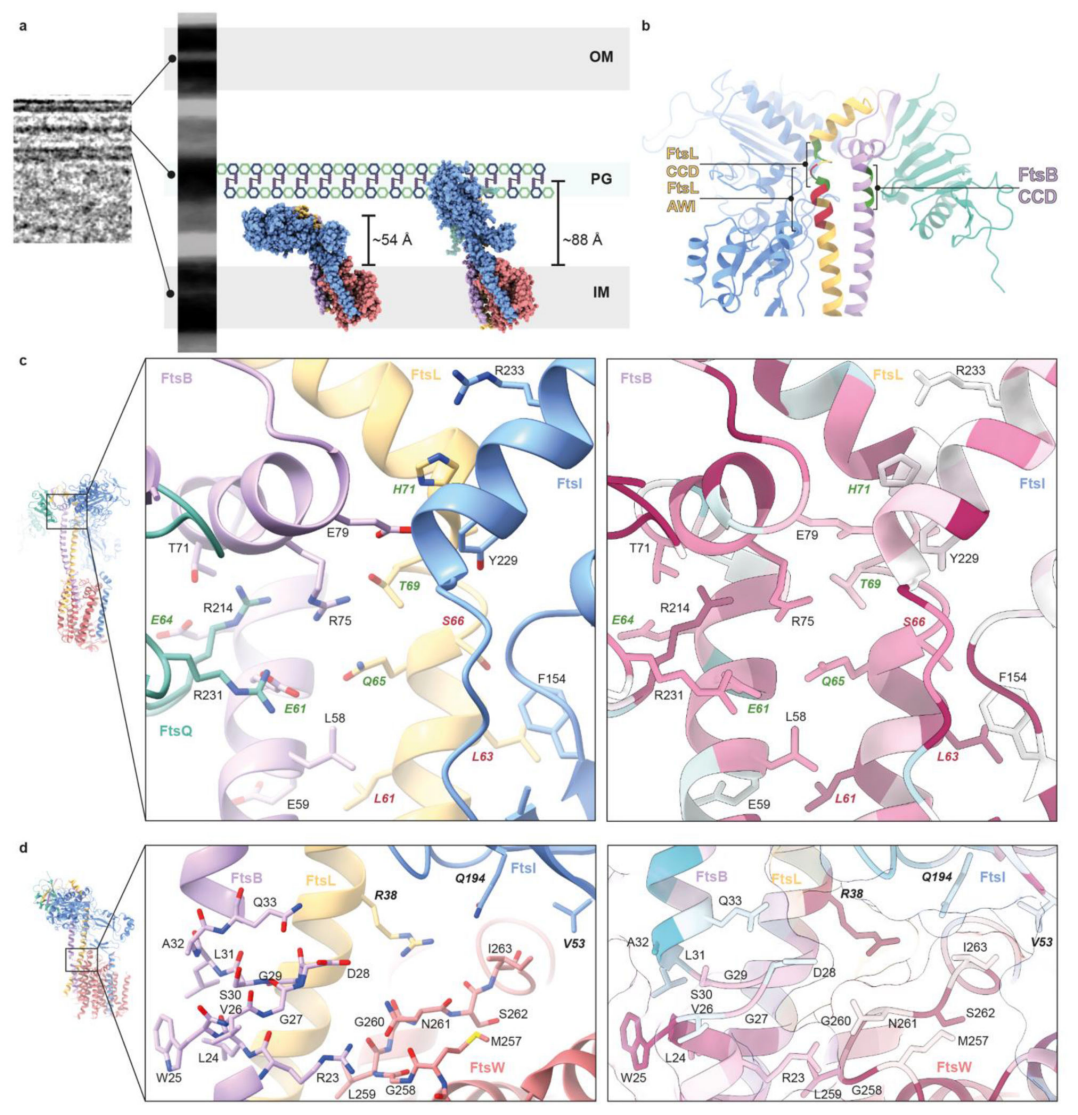
Interactions that affect divisome regulation. **a)** To-scale model of the *E. coli* cell envelope generated from a cellular electron cryotomogram. The cryo-EM FtsWIQBL atomic model (reported here, left) and the AlphaFold 2 prediction (right) are docked into the inner membrane. When placing both structures into the inner membrane, the FtsI transpeptidase domain of our cryo-EM structure does not extend to the peptidoglycan layer, yet it does so in the AlphaFold 2 structure. The measured distances of the active site residue FtsI^S294^ to the inner membrane plane are indicated. **b)** The C-termini of the FtsB and FtsL coiled coils with highlighted Constriction Control Domain (CCD, green) and Activation of FtsWI (AWI, red) regions, showing the FtsB^CCD^ facing FtsQ and FtsL^AWI^ facing FtsI. **c)** Region around the activating mutations FtsL^Q65^ (E88 in *E. coli*) and FtsB^E61^ (E56 in *E. coli*). Residues with known divisome activating mutations, which lead to smaller cells, are shown in green. Residues with known loss-of-function mutations, which cause a defect in cell division, are shown in red. Sequence conservation analysis [calculated with ConSurf^61^] shows a high degree of conservation for many residues in this area. **d)** Residues surrounding the region of discontinuity in the FtsB coiled coil. This region contains residues with known loss-of-function mutations (shown in bold). Many residues in this region are highly conserved, including FtsL R38 (R61 in *E. coli*), which inserts between FtsI and FtsW and is located close to a highly conserved loop in FtsW (M257-I263, Q279-V285 in *E. coli*) that is in close proximity to the putative active site residue D275 (D297 in *E. coli*).

We used AlphaFold 2 multimer^24^ (AF2) to predict *Pa*FtsWIQBL and many large-scale and fine features observed in the *Pa*FtsWIQBL structure were predicted correctly by AF2, including the lack of an interaction between the membrane-embedded FtsQ™ and FtsWIBL™. However, in the AF2 model the periplasmic FtsIQBL interaction site is rotated upwards by about 30°, moving FtsI^TP^ closer towards where the peptidoglycan layer is located (Extended Data Fig. 7a, c). Furthermore, a small rearrangement in the anchor subdomain FtsI^pedestal^-FtsL^α1^ shifts the interacting residues on FtsI^pedestal^ from 208-212 to 203-206. To understand the implications of these differences, both structures were fitted into a to-scale model of the cell envelope of *E. coli*, produced from a cellular electron cryo-tomogram (Fig. 3a). Using the cryo-EM structure, the active site of FtsI^TP^ does not reach the peptidoglycan layer, but does so in the more extended AF2 model. Since AF2 uses evolutionary couplings between amino acids^24^, in addition to protein structural features that correlate with sequence, it more likely predicts the active state of FtsWIQBL that one would expect to be selected for during evolution. Thus, the cryo-EM structure and AF2 prediction may represent the inactive and active (catalytic) states of the divisome core complex, respectively. Since our sample shows transglycoylase activity in vitro and the cryo-EM structure is substrate-free (apo), it is at least theoretically possible that Lipid II substrate binding contributes to the interconversion of the two states. In addition, other factors such as FtsN or peptidoglycan chains might be required to achieve the proposed conformational change. Recently, a study on RodA-PBP2 reported a similar upswinging mechanism^25^, which indicates that the concept of regulating the enzymatic activities via restricting access to the PG layer might be conserved between the divisome and the elongasome. However, significant differences exist between divisome and elongasome regarding the conformation before the upswinging motion and most likely also between the signals required to initiate this conformational change.

FtsN has been reported to trigger constriction in cells^26,27^. It is the last protein to be recruited to the division site in *E. coli* and its recruitment is dependent on the presence of earlier divisome proteins, including FtsA, FtsQ and FtsI^26,28–30^. However, we have not been able to generate a biochemically stable *E. coli* FtsN-FtsWIQLB complex and previous studies reported that the addition of the FtsN periplasmic domain did not yield an increase in *P. aeruginosa* TG activity *in vitro*^7^. Whether FtsN activates the core divisome beyond the TG activity levels seen here through binding of FtsQLB, or whether the *in vitro* sample cannot be further activated will need further investigations including addition of other divisome components, e.g. FtsA, FtsN and/or DedD, as well as the substrate Lipid II. It is also possible that FtsN is involved only in regulating TP activity.

### Interactions that affect divisome regulation

The Constriction Control Domain (CCD) of the divisome was identified previously from a set of mutations that allow partial or complete bypass of the requirement for FtsN in *E. coli*^31,32^. In our structure, these residues cluster at the top of FtsL^α1^ and FtsB^α2^, with the FtsB CCD mutations facing FtsQ^β^. Furthermore, in close proximity are the Activation of FtsWI (AWI) residues on FtsL^α1^ that display a dominant-negative phenotype when mutated^19^ (Fig. 3b). The CCD residues FtsB^E61^ (*Ec*E56G/A/K/V/H) and FtsL^Q65^ (*Ec*E88K/V) point towards a positively charged cavity formed by three arginine residues (FtsQ^R214^, FtsQ^R231^, FtsB^R75^), flanked by FtsL^T69^ (*Ec*G92D, CCD residue) on one side and FtsB^E64^ (*Ec*D59V, CCD residue) and FtsB^T71^ on the other side. This interface contains many charged and conserved residues (Fig. 3c), and removal of a charge or introduction of the opposite charge could well result in destabilisation of the interface and potentially increased flexibility of the protein. This may allow FtsWIQBL to more readily adopt an elongated, a more active conformation, as possibly indicated by the AF2 model, and with less or no activation signal, for example from FtsN.

A dominant negative phenotype was previously reported for the AWI mutation *Ec*L86F^19^ and its *P. aeruginosa* equivalent FtsL^L63^ interacts with FtsI^F154^ in FtsI^pedestal^ (Fig. 3c). Replacing the leucine with the bulkier phenylalanine likely causes a steric clash that weakens the FtsI-FtsL interaction. FtsL^S66^ (*Ec*N89S, *Pa*S66D) was classified as a CCD mutation in *E. coli*^31^, but has a dominant negative phenotype in *P. aeruginosa*, with reduced *in vitro* TG activity^7^, likely due to a steric clash with FtsI^F154^. Mutation of FtsL^L61^ (*Ec*L84K/D) has a dominant-negative phenotype and affects FtsL localisation to the septum^19,33^, indicating that the integrity of the FtsBL coiled-coil interaction is vital for a functional complex, as is well supported by our structure where the FtsBL coiled coil is at the centre of the complex.

The AWI residue FtsL^R38^ (*Ec*R61C) is highly conserved and located between FtsW^M257-I263^ and the anchor subdomain of FtsI^pedestal^ (Fig. 3d). Mutation of this residue causes a dominant-negative phenotype in both *E. coli* and *P. aeruginosa* and reduced *in vitro* TG activity in *P. aeruginosa*^7,19^. FtsL^R38^ might interact with the highly conserved FtsW^G260^ and FtsW^S262^ residues and stabilise FtsW^M257-I263^ together with FtsB^R23^, a hypothesis supported by the fact that the corresponding loop in RodA and RodA-PBP2 is disordered^18,23^. The aforementioned FtsB coiled-coil discontinuity, C-terminal of FtsB^R23^, might be required to allow for some flexibility of FtsB during the catalytic cycle of FtsW. *Ec*FtsI^L62P^ and *Tt*PBP2^L43R^ correspond to *Pa*FtsI^V53^ and result in a strong cell division defect and reduced TG activity *in vitro*, respectively^18,34^. *Pa*FtsI^V53^ interacts with *Pa*FtsW^I257^ in our structure, bringing the linker between FtsI™ and FtsI^pedestal^ in close proximity to FtsW. Thus, FtsW^M257-I263^ presents an interaction site for FtsL, FtsB and FtsI in close proximity to the putative FtsW active site.

**Extended Data Fig. 8:**
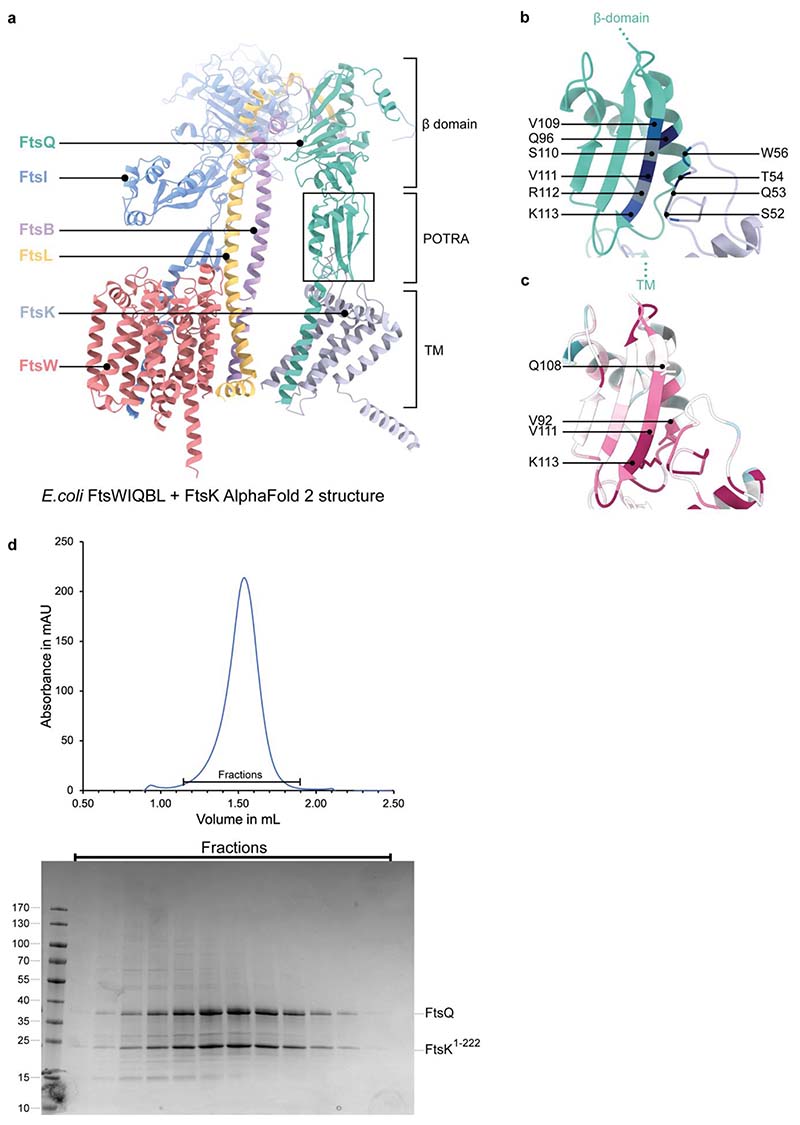
Interactions of FtsQ with FtsK. **a)** AlphaFold2 model of *E. coli* FtsWIQBL + FtsK N-terminal domain (residues 1-222; FtsK^1-222^, grey), showing a predicted interaction between FtsQ^POTRA^ and a periplasmic loop from FtsK (FtsK^W51-H57^). **b)** Co-evolutionary coupling analysis calculated with EVcouplings^36^ finds six out of the ten residue pairs located between FtsQ^POTRA^ and FtsK^W51-H57^ in the AF2 model in a): FtsQ^V109^ – FtsK^W56^ (blue), FtsQ^Q96^ – FtsK^T54^ (dark blue), FtsQ^S110^ – FtsK^Q53^ (gray), FtsQ^V111^ – FtsK^T54^ (dark blue), FtsQ^R112^ – FtsK^Q53^ (grey), and FtsQ^K113^-FtsK^S52^ (blue). **c)** Sequence conservation analysis (calculated using ConSurf webserver^61^) of the same area shows that the β-strands of FtsQ and FtsK^W51-H57^ that are predicted to interact are highly conserved. Amino acid residues that abolish FtsQ localisation (which is dependent on FtsK septum localisation in cells) when mutated are shown as sticks and are labelled^10^. **d)** Size-exclusion trace and SDS-PAGE gel of the co-expression and purification of *E. coli* FtsQ and FtsK^1-222^ shows clear co-migration of the proteins.

The recruitment of FtsQ to midcell requires FtsK^10,29,35^. AF2 predicts that the interaction between FtsK^1-222^ and FtsWIQBL, occurs through FtsQ^POTRA^ β2 and α3; this interaction is also identified by coevolutionary coupling analysis using EVcouplings^36^ as an FtsQ-FtsK interaction hotspot (6/10 couplings, Extended Data Fig. 8a, b). Residues previously identified as impairing FtsK recruitment when mutated^10^ (*Ec*Q108, *Ec*V92, *Ec*V111, *Ec*K113) map onto this conserved region (Extended Data Fig. 8c). We copurified an FtsQK^1-222^ complex using *E. coli* proteins, which confirms that the two proteins interact tightly and constitutively (Extended Data Fig. 8d). To further our understanding of divisome recruitment and regulation, in the future larger divisome complexes will need to be assembled. For example, divisome interactions with FtsA, through FtsN, FtsK and possibly FtsQ have the potential to modify the conformations, oligomeric states and activities of the core complex and its enzymes.

We report the structure of the essential bacterial cell division complex and important future antibiotic target FtsWIQBL from *Pseudomonas aeruginosa* and show that *Pa*FtsWIQBL forms a stable Y-shaped complex that harbours intrinsic TG activity. Our *Pa*FtsWIQBL structure is able to explain many subunit contacts that have previously been shown to be important through loss-of-function and bypass mutations. In addition, an AF2 model reveals a different, likely catalytically competent state that allows for peptidoglycan crosslinking by FtsI. While our analysis hints at the nature of the catalytic state, further research is needed to resolve more states and their associated conformation changes, which possibly requires the addition of activating proteins such as FtsA and/or FtsN as well as the substrate Lipid II and its derivatives or products as ligands. It will be particularly exciting to resolve the enzymatic mechanism of the FtsW TG since it is a promising drug target for novel antibiotics given its function, conservation, periplasmic accessibility and very wide phylogenetic distribution. To gain a deeper understanding of the mechanism of the divisome, the inclusion of upstream and downstream proteins e.g., FtsEX, FtsK, PBP1b, and DamX will also be necessary. Our work is an important milestone in the 25-year quest for a molecular understanding of the ancient, near ubiquitous and medically important process of FtsZ-based bacterial cell division.

## Materials and methods

### Cloning

Primers used for Gibson assembly are listed in Supplementary Table S2. The generated expression plasmids are listed in Supplementary Table S3. All constructs were confirmed by sequencing.

#### P. aeruginosa FtsWIQLB (PaFtsWIQBL)

Cloning, expression and purification of *Pa*FtsWIQBL (Uniprot: Q9HW00 (FtsW), G3XD46 (FtsI), G3XDA7 (FtsQ), Q9HVZ6 (FtsL), Q9HXZ6 (FtsB)) were adapted from a previously published protocol^7^ with some changes, including the switch from a FLAG-tag to a Strep-tag.

#### E. coli FtsWIQLB (EcFtsWIQBL)

The *Ec*FtsWIQLB complex (Uniprot: P0ABG4 (FtsW), P0AD68 (FtsI), P06136 (FtsQ), P0AEN4 (FtsL), P0A6S5 (FtsB)) was expressed in insect cells through a single baculovirus vector, which was assembled using the biGBac system^37^. To ensure equal expression levels, a fusion of FtsW and FtsI, as is found naturally in some organisms (Supplementary Table S4), was created with a GSGASG cytoplasmic linker between the FtsW C-terminus and FtsI N-terminus.

#### E. coli FtsQK^1-122^ (EcFtsQK^1-122^)

Genes encoding FtsQ-His_6_ (Uniprot P0613) and TwinStrep-FtsK^1-222^ (Uniprot P46889) were ordered as gBlocks (IDT) and cloned into pLIB. The gene expression cassettes were amplified and inserted into pBIG1a^37^ forming pNJ069.

### Baculovirus generation

Baculoviruses containing pFE758 and pNJ069 were used for insect cell expression of *Ec*FtsWIQLB and *Ec*FtsQK^1-222^ respectively. Recombinant baculoviral genomes were generated by TN7 transposition in DH10bacY cells^39^ and this bacmid was used to transfect Sf9 cells (Thermo Scientific) using FuGENE (Promega). After 3-5 days, the culture was centrifuged and the virus-containing supernatant was harvested and stored in the presence of 1% fetal bovine serum (FBS).

### Bacterial expression

*Pa*FtsWIQLB was expressed in *E. coli*. pLK1, pLK2 and pLK3 were sequentially transformed into *E. coli* C43(DE3). 120 mL of overnight culture were added to 12 L TB media with 2 mM MgCl_2_, kanamycin (25 µg/mL), chloramphenicol (25 µg/mL) and ampicillin (50 µg/mL), and grown at 37°C to an OD_600_ of 0.7. Protein expression was induced with 1 mM IPTG and 1 g arabinose/L and continued at 18°C overnight. Cells were harvested by centrifugation for 20 min at 4,000 g at 4°C, flash frozen in liquid nitrogen and stored at -80°C.

### Insect cell expression of *Ec*FtsWIQBL and *Ec*FtsQK^1-222^

Sf9 cells were grown in Insect-Xpress medium (Lonza) to a density of 1.5-2 million cells/ml, infected with ~1% amplified baculovirus and harvested by centrifugation after 60-70 h, when cell viability reached ~80%. Cell pellets were washed with phosphate buffered saline (PBS), flash frozen in liquid nitrogen and stored at -80°C.

### Protein purifications

All purifications, including centrifugation steps, were performed at 4°C. Proteins were aliquoted, flash frozen in liquid nitrogen and stored at -80ºC, except when cryo-EM grids were directly prepared from freshly eluted protein. For the activity assay (see below) protein concentration was determined using Bio-Rad Protein Assay dye reagent concentrate (Bio-Rad).

#### PaFtsWIQBL

Cells from 12 L of *E. coli* culture were resuspended in a final volume of 300 ml Lysis Buffer (20 mM HEPES, 150 mM NaCl, 20 mM MgCl_2_, pH 7.5, 1 mM DTT) containing DNase (Sigma) and RNase (Sigma) and twice passed through a cell disruptor (Constant Systems) at 25,000 psi. The lysate was centrifuged for 1 h at 234 998 g (Type 45 Ti rotor, Beckmann). Membranes were homogenised using a dounce tissue grinder (Whaeton) in Solubilisation Buffer (20 mM HEPES, 500 mM NaCl, 20 % glycerol, pH 7.0). Membrane-bound proteins were solubilised with Lauryl maltose neopentyl glycol detergent (LMNG, Anatrace) at a final concentration of 1% (w/v) and rotated for at least 1 hour. The volume was doubled with Solubilisation Buffer and 12 μl benzonase (Merck) were added. The solution was centrifuged for 1 h at 234 998 g (Type 45 Ti rotor, Beckman) and the supernatant was bound to 2 mL (CV) of equilibrated Strep XT4 Flow beads (IBA) while rotating for 2 h. Beads were collected in a column and washed with 50 mL of Wash Buffer 1 (Solubilisation Buffer plus 0.1% LMNG) and 50 ml of Wash Buffer 2 (Solubilisation Buffer plus 0.01% LMNG). Protein was eluted from the Strep beads over 10 CV in 2 mL fractions with Elution Buffer (20 mM HEPES, 500 mM NaCl, 20% glycerol, 50 mM biotin, pH 7.0, 0.005% LMNG). All elution fractions were pooled and concentrated to 50 μL using 100 kDa centrifugal concentrators (Vivaspin). The concentrated sample was further purified using a Superose 6 Increase 3.2/300 size-exclusion column (Cytiva), equilibrated in Size Exclusion Buffer (20 mM HEPES, 300 mM NaCl, pH 7.0, 0.005% LMNG).

#### EcFtsWIQBL

Sf9 Cells were resuspended in Lysis Buffer (20 mM HEPES pH 8.0, 500 mM NaCl, 10% glycerol, 2 mM TCEP), containing 1 mM PMSF, protease inhibitor tablets (cOmplete EDTA-free PI (Roche) 1 per 25 mL), DNase (Sigma), RNase (Sigma) and sonicated for 2 min (1 s pulse on, 10 s pulse off, 70% intensity), after which 2 mM EDTA was added. The lysate was centrifuged for 1 h at 235,000 g. The pellet was homogenised using a dounce tissue grinder (Whaeton) in Solubilisation Buffer (20 mM HEPES pH 8.0, 10 mM MgCl_2_, 500 mM NaCl, 20% glycerol), containing 1 mM PMSF, and PI tablets. Detergent glyco-diosgenin (GDN, Anatrace) was added to a final concentration of 1% to solubilise the membrane-bound proteins and rotated for 2 h, before centrifugation to remove nuclei (10 min, 3,200 g). The supernatant was 5-fold diluted with Strep Buffer (20 mM HEPES, pH 8.0, 350 mM NaCl, 10% glycerol, 1 mM TCEP) in the presence of Benzonase (Merck) and the NaCl concentration reduced to 350 mM before centrifuging for 45 min at 142,000 g. The supernatant was cycled over a 5 mL StrepTrap-HP column (Cytiva) overnight. The StrepTrap-HP column was washed with 50 column volumes of Strep Buffer including 0.01 % GDN at 5 mL/min. The protein complex was eluted in Strep Buffer supplemented with 2.5 mM desthiobiotin (Sigma) at 1 mL/min. Fractions containing the complex were pooled and concentrated to 50 µL using 100 kDa centrifugal concentrators (Vivaspin) and further purified using a Superose 6 Increase 3.2/30 size-exclusion column (Cytiva) in SEC buffer (20 mM HEPES pH 8.0, 350 mM NaCl, 10% glycerol, 1 mM TCEP, 0.01% GDN).

#### EcFtsWIQBL reconstitution in nanodiscs for cryo-EM

The nanoquick protocol^40^ was adapted to reconstitute the complex into nanodiscs. Proteins were expressed and the membrane was solubilised as described above but instead of GDN a mixture of LMNG:CHS (10:1) was used at a final concentration of 1%. The solubilised protein was incubated overnight with StrepTactin sepharose beads (Cytiva). The beads were washed in Strep Buffer including 0.1% LMNG:CHS (10:1) and then into SEC buffer including 2% glycerol and 0.1 % LMNG:CHS (10:1). A thousand fold molar excess of POPG was added and incubated for an hour, followed by a 20-fold excess of MSP2N2 (purified as described in^41^). After 15 min, activated SM-2 BioBeads (BioRad) were added and the mixture was rotated overnight. The protein complex was eluted in Strep Buffer supplemented with 2.5 mM desthiobiotin (Sigma) and subjected to size-exclusion chromatography as above in SEC buffer without detergent.

#### EcFtsQK

Cells from 1 L of culture were resuspended in 50 mL Lysis Buffer (50 mM Tris, 500 mM NaCl, pH 8.0) supplemented with DNase (Sigma), RNase (Sigma), and protease inhibitor tablets (cOmplete EDTA-Free PI (Roche), 1 per 25 mL) and sonicated for 2 min (1 sec on, 10 sec off, 70% intensity). The lysate was centrifuged for 25 min at 25,000 g (25.50 rotor, Beckmann) and the supernatant subsequently for 1 h at 200,000 g (Ti 45 rotor, Beckmann). The membranes were homogenised with a Dounce homogeniser and solubilised in 35 mL Solubilisation Buffer (50 mM Tris, 350 mM NaCl, pH 8.0, 1% GDN, 10% glycerol) for 2 h. The solution was diluted to 50 mL with Dilution Buffer (50 mM Tris, 350 mM NaCl, pH 8.0, 10% glycerol) and centrifuged for 30 min at 80,000 g (Ti 75 rotor, Beckmann). The supernatant was diluted to 500 mL with dilution buffer and cycled overnight over a 1 mL StrepTrap-HP column (Cytiva). The column was washed with 70 mL of buffer A1 (50 mM Tris, 350 mM NaCl, 0.006% GDN, 10% glycerol, pH 8.0) and the complex eluted with 20 mL of Buffer A2 (50 mM Tris, 350 mM NaCl, 0.006% GDN, 10% glycerol, 2.5 mM desthiobiotin, pH 8.0) in 2 mL fractions. The fractions containing FtsQK were pooled, bound to an equilibrated 1 mL HisTrap (Cytiva) column and eluted using a step gradient from Buffer B1 (50 mM Tris, 350 mM NaCl, 0.006% GDN, 10% Glycerol, 20 mM imidazole, pH 8.0) to Buffer B2 (50 mM Tris, 350 mM NaCl, 0.006% GDN, 10% glycerol, 1 M imidazole, pH 8). The elution was concentrated to 50 μL using a 100 kDa cutoff centrifugal concentrator (Vivaspin) before further purification using a Superose 6 Increase 3.2/300 size-exclusion column in SEC buffer (50 mM Tris, 100 mM NaCl, 0.006% GDN, 10% glycerol, pH 8.0).

#### S. aureus PBP4 (SaPBP4)

His-tagged *Sa*PBP4^21-383^ was expressed as described previously^42^ and purified as follows. Cells were lysed in Buffer A (50 mM Tris pH 7.5, 500 mM NaCl) containing 1 mM PMSF, DNase, RNase and PI tablets using a cell disruptor (Constant Systems) at 25 kpsi. The lysate was centrifuged at 100,000 g for 30 min. The supernatant was supplemented with 1% Buffer B (Buffer A + 1 M imidazole, pH 7.5) and cycled twice over a 5 mL HisTrap-HP column (Cytiva). The column was washed with 150 mL 1% B, 550 mL 2% B and eluted in steps of 5%, 30% and 50% B. Fractions containing *Sa*Pbp4 were concentrated by centrifugal filtration (Vivaspin) and further purified over a Superdex 200 PG 16/60 size-exclusion column (Cytiva) in SEC buffer (20 mM MES pH 6.0, 300 mM NaCl). Monomer peak fractions were combined and concentrated to 52 g/L by centrifugal filtration (Vivaspin), then flash frozen in aliquots before being stored at -80°C.

### Cryo-EM single particle structure determination

#### Grid preparation PaFtsWIQBL

Grids were prepared with freshly purified protein from the peak fraction of the SEC elution at a final concentration of 1.2-1.3 g/L, diluting with SEC buffer if necessary. 3 μL of sample were pipetted onto a glow discharged (PELCO easiGlow, 25 mA, 45 sec) 300 mesh Cu 0.6/1 grid (Quantifoil) and blotted for 4 s at blot force 4, 100% humidity and 4°C, before plunge-freezing in liquid ethane using a Vitrobot Mark IV (Thermo Fisher Scientific, TFS).

#### Grid preparation EcFtsWIQBL

Grids were prepared with freshly purified protein (*Ec*FtsWIQBL reconstituted in nanodiscs) from the peak fraction of the SEC elution at a final concentration of 0.5 g/L, diluted with SEC buffer if required. 3 μL of sample were pipetted onto a freshly glow-discharged (PELCO easiGlow, 30 mA, 60 sec) 300 mesh UltraAu 0.6/1 grid (Quantifoil). After a 20 s wait time the grids were blotted for 2.5 s at strength 5, 100% humidity and 10°C, before plunge-freezing in liquid ethane using a Vitrobot Mark IV (Thermo Fisher Scientific, TFS).

#### Data collection PaFtsWIQBL

Data from 5531 micrographs were collected on a Titan Krios G3 (TFS) equipped with a Gatan K3 camera and a Gatan Quantum energy filter (20 eV slit width). TFS’s EPU software was used to collect the micrographs with fringe-free imaging in counting mode at a nominal pixel size of 1.09 Å. The exposure time was 2.5 sec at a dose of 21 e/px/s, defocus between -1.2 and -3 μm, and 40 fractions per micrograph were collected.

#### Data collection EcFtsWIQBL

Data from 2794 micrographs were collected on a Titan Krios G3 (TFS) equipped with a Falcon 4 camera. TFS’s EPU software was used to collect the micrographs in counting mode at a nominal pixel size of 1.1 Å. The total dose was set to 39 e/Å^2^ and the defocus between -1.2 and -2.8 μm.

#### Data processing

Unless stated otherwise, all processing was done in RELION 4.0^43^. Motion correction was performed using RELION’s own implementation of the MotionCor2 algorithm^44^ with 5x5 patches. CTFFind-4.1^45^ was used for CTF estimation. The initial reference was generated in CryoSPARC^46^ from a different dataset of the same sample (not used for the final reconstruction). Particles were picked with Topaz^47^ 7,276,623 particles total, (1,398 per micrograph on average) and extracted 4x binned with a boxsize of 70 px and a pixel size of 4.36 Å/px. The particles were split into seven subsets for initial 3D classification into three classes. The best classes of each job were selected and refined. The refined particles were combined into two sets of particles and subjected to 3D classification without alignment. The best classes from each alignment were selected (1,997,326 and 2,922,553 particles total), combined and subjected to a 3D refinement. A mask (extended by 2 px and added soft edge of 2 px) was used to subtract the micelle density from the complex. The subtracted particles were subjected to a 3D classification without alignment and two classes were selected (344,300 and 461,499 particles), re-extracted (2x binned with a boxsize of 140 pix and a pixel size of 2.18 Å/px) and subjected to 3D refinement. 3D classification without alignment was run and the best class (160,615 particles) was selected. The particles were re-extracted (no binning, 280 pix, 1.09 Å/px) and subjected to one round of polishing, 3D refinement, CTF refinement and 3D refinement. Following subtraction of the micelle the particles were subjected to a round of 3D refinement and 3D classification without alignments. From this 3D classification, the best class was selected (136,364 particles) and 3D refined two times, the second time using a mask that excluded the POTRA domain of FtsQ. After postprocessing with the calibrated pixel size (see below) the final reconstruction had an overall resolution of 3.7 Å as determined by Fourier shell correlation (FSC, cutoff 0.143).

#### Model building

The pixel size was calibrated using Chimera^48^ and a published crystal structure of *Pa*FtsI (PDB:3OCN) and subsequently adjusted to 1.05 Å/px during postprocessing in RELION. This map was used to fit a model of *Pa*FtsWIQBL predicted with AlphaFold2^24,49^ and manually adjusted using MAIN^50^ and Coot^51^ (Version: 0.9.8.3), and real-space refined using Phenix^52^ (Version: 1.19.2-4158). Figures were prepared using ChimeraX-1.4^53^.

### Lipid II extraction

Lipid II was extracted from *Enterococcus faecalis* (DSMZ 2570) as described before^54^. Briefly, an overnight culture of *E. faecalis* was diluted 1:100 into 3 L of Brain heart infusion (BHI, Merck) and grown at 37°C, 180 rpm to an OD_600_ of 0.7. Vancomycin (Sigma) and moenomycin (Santa Cruz) were added at 10 µg/mL and 5 µg/mL, respectively, and after 20 min cells were centrifuged in pre-cooled bottles at 4,500 g for 20 min. Cell pellets were resuspended in BHI, centrifuged in Falcon tubes at 3,200 g for 10 min, flash frozen and stored at -20°C overnight. Frozen cell pellets were thawed in a total of 30 mL phosphate-buffered saline (PBS), divided equally into two 250 mL glass Erlenmeyer flasks and 17.5 mL chloroform and 35 mL methanol were added. After 2 h of vigorously stirring at room temperature, the mixture was spun in Teflon tubes for 10 min at 4,000 g at 4°C and the supernatant from each Erlenmeyer was combined with 30 mL chloroform and 22.5 mL PBS. The mixture was stirred vigorously for 2 h at room temperature, then spun for 10 min at 4,000 g at 4°C. The tubes were left at room temperature for 1 h until the supernatant was clear. The interface was then transferred with a glass Pasteur pipette to a 25 mL separatory funnel and left to settle overnight at 4°C. The lower organic phase was discarded and the interface dried in a 25 mL round bottom flask on a rotary evaporator at 40°C. The dried interface was resuspended in 7.5 mL pyridinium acetate:n-butanol (1:2) (PB) and 7.5 mL n-butanol-saturated water. Pyridinium acetate was prepared by adding 51.5 mL glacial acetic acid dropwise to 48.5 mL pyridine and filtered before use. The Lipid II extract was transferred to a 25 mL separatory funnel and the bottom phase was re-extracted with 5 mL PB. The top phase from the re-extraction was added to the top phase in the separatory funnel and extracted three times with 5 mL n-butanol-saturated water. The top phase was dried using a rotary evaporator at 40°C and resuspended in chloroform:methanol (1:1), partially dried under a stream of nitrogen gas and then transferred to a 250 µL non-stick glass vial (Agilent), in which it was dried completely. This was repeated 4 times to ensure efficient transfer before resuspending the Lipid II extract in 210 µL chloroform:methanol (1:1). The Lipid II extract was assessed by spotting 1-2 µL on a HPTLC silica gel 60F254 plate (Merck). The TLC plate was developed in a mixture of chloroform:methanol:water:ammonia (88:48:10:1) and Lipid II was visualised by heating the plate after soaking in phosphomolybdic acid (PMA) as described previously^55^.

### Deprotection of FMOC-BDL

D-Lys-Biotin (BDL) was prepared following a standard deprotection protocol^42^. Briefly, 15 mg Fmoc-D-Lys(Biotin)-OH (Santa Cruz Biotechnology) was stirred in 3.1 mL of 20% piperidine/dimethylformamide and 466 µL toluene for 40 min at room temperature, then dried in vacuum at 50°C. The sample was resuspended in 5 mL water, stirred for 2 h at room temperature and then filtered through a 0.22 µm filter. The filtrate was pipetted into a tared tube, frozen on dry ice and then freeze dried. The residue was dissolved in water to make a 10 mM stock, aliquoted and stored at -20°C.

### Transglycosylase activity assay

Lipid II used to monitor glycosyltransferase activity of the protein complex was dried using a nitrogen stream and dissolved in an equal volume of dimethyl sulfoxide (DMSO). The reaction and detection of glycan strands was adopted from previously published protocols^3,23,42,56^. Briefly, *Pa*FtsWIQLB and *Ec*FtsWIQLB were mixed at a final protein concentration of 1 µM in 10µL with 1 µL 10x Reaction Buffer (500 mM Tris pH 7.5, 200 mM MnCl_2_), 1 µL DMSO and 1 µL Lipid II and incubated for 30 min at 25°C. Proteins were heat inactivated for 2 min at 95°C. Lipid II and glycan strands were labelled by incubating the reaction with 26 µM *Sa*Pbp4 and 20 mM BDL for 1 h at 25°C. An equal volume of Laemmli SDS-PAGE buffer was added and the mixtures were heat inactivated for 3 min at 95°C. Glycan strands were separated from Lipid II on a 4-20% Criterion TGX polyacrylamide gel (Bio Rad), run for 45 min at 200 V. After blotting onto PVDF membrane, the blot was incubated for 2 h in Superblock blocking buffer TBS (Thermo Scientific), followed by incubation with a 1:5000 dilution of IRDye800CW Streptavidin (LI_COR Bioscience) in TBS buffer at room temperature for 1 h. The blot was washed three times in PBS buffer and bands were visualised using an Odyssey CLx imaging system (Li-COR Bioscience).

### Electron cryo-tomography of *E. coli* cells

A culture of *E. coli* strain B/r H266 expressing plasmid pRBJ212^57^ was grown in LB media at 37°C, 180 rpm to an OD_600_ of 0.6. Cells were concentrated 10x by centrifugation and mixed with 10 nm protein A gold fiducials in a 1:10 ratio. 4 µL of this mixture was applied to a 200-mesh Cu2/2 grid (Quantifoil), back-blotted and plunge-frozen in liquid ethane using a manual plunger. Cells were thinned by cryo-focused ion beam milling (cryoFIB) using a Scios dual beam FIB-SEM (TFS). Before milling, grids were sputtered with a protective layer of organic platinum using the gas injection system. Lamellae were milled in a stepwise fashion, gradually reducing the beam current as the lamellae were thinned, from 1 nA to 30 pA and at a nominal milling angle of 10°. CryoET was carried out on a Krios microscope (ThermoFisher) equipped with a Gatan imaging filter and K2 camera. Tilt series were collected using serialEM software^58^, using a bidirectional tilt scheme from -10° (to flatten the lamella) with a 2° increment and a total dose of 112 e^-^/Å^2^, divided over 56 images, each with 10 frames. The pixel size was 3.7 Å and the defocus target was -5 µm. Frame alignment and tilt series alignment were performed using IMOD^59^, 2x binned aligned tilt series were generated and used to generate a SIRT reconstruction using tomo3D^60^, which were then low-pass filtered to 20 Å.

## Extended Data

## Supplementary Material

Morph.mp4

Movie 1_2

Plasmid Maps

Supplementary Tables

## Data Availability

The final cryo EM map has been deposited in the Electron Microscopy Data Bank (EMDB) with the accession code EMD-16042. The final model has been deposited with the Protein Data Bank (PDB) with the accession code 8BH1. PDB entries 6H9N, 3PBN, 5Z2W, 6BAS, 6PL5, 3OCN were used for structural superposition, analyses and pixel size calibration. Source data are provided with this paper. Uniprot accession codes for the purified complexes: *Pa*FtsWIQBL (Uniprot: Q9HW00 (FtsW), G3XD46 (FtsI), G3XDA7 (FtsQ), Q9HVZ6 (FtsL), Q9HXZ6 (FtsB)) *Ec*FtsWIQBL (Uniprot: P0ABG4 (FtsW), P0AD68 (FtsI), P06136 (FtsQ), P0AEN4 (FtsL), P0A6S5 (FtsB)) *Ec*FtsQK (Uniprot: P0613 (FtsQ), P46889 (FtsK))
