# Supplementary material for: Cryo-EM structure of the bacterial divisome core complex and antibiotic target FtsWIQBL
Source: Nat Microbiol. Author manuscript; Available in PMC 2023 Jun 2. (PMC7614612; doi:10.1038/s41564-023-01368-0)
Supplement: Supplementary Tables [file EMS174341-supplement-Supplementary_Tables.docx]

**Supplementary Table S1: Imaging statistics cryo-EM**

| **Data collection and processing** | ***Pa*FtsWIQBLEMD-16042, PDB 8BH1** |
| --- | --- |
| Magnification | 81,000x |
| Voltage (kV) | 300 |
| Electron fluency (e^-^/Å^2^) | 41 |
| Defocus range (μm) | -1.2 to -3 |
| Pixel size (Å) | Nominally 1.09, refined to 1.05 |
| Symmetry | C1 |
| Initial particles (number) | 7,276,623 |
| Final particles (number) | 136,364 |
| Map resolution (Å) | 3.7 |
| FSC threshold | 0.143 |
| **Model** |  |
| Initial model used | AlphaFold2 model |
| Model resolution (Å) | 3.9 |
| FSC threshold | 0.5 |
| Map sharpening B factor (Å^2^) | -127 |
| *Model composition* |  |
| Non-hydrogen atoms | 9,269 |
| Protein residues | 1,195 |
| RMSD bond lengths (Å) | 0.003 |
| RMSD bond angles (°) | 0.665 |
| *Validation* |  |
| MolProbity score | 1.76 |
| Clashscore | 12.23 |
| Rotamers outliers (%) | 0.0 |
| Ramachandran plot |  |
| Favored (%) | 97.12 |
| Allowed (%) | 2.88 |
| Disallowed (%) | 0 |

**Supplementary Table S2: Primers.**

| **Name** | **Sequence 5’-3’** |
| --- | --- |
| Ndeintoplib.for | ATGCGGTCCGAAGCGCGC |
| Ndeintoplib.rev | ATGCGGATCCGCGCCCGATG |
| MTAstrep.for | GGGCGCGGATCCGCAATGACAGCATGGTCACATCCGCAGTTTG |
| WlinkerI.rev | TCGCCGCTGCTTTCATACCTGATGCGCCGCTTCCTCGTGAACCTCGTACAAACG |
| CasI.for | AACGCTCTATGGTCTAAAGATTTAAATCGACCTACTCCGGAATATTAATAGATC |
| CasI.rev | AACGCTCTATGGTCTAAAGATTTAAATCGACCTACTCCGGAATATTAATAGATC |
| CasII.for | AAACTGGATACTATTGCACGTTTAAATCGACCTACTCCGGAATATTAATAGATC |
| CasII.rev | AAACATCAGGCATCATTAGGTTTATTTAAATGGTTATGATAGTTATTGCTCAGCG |
| CasIII.for | AAACCTAATGATGCCTGATGTTTAAATCGACCTACTCCGGAATATTAATAGATC |
| Casv.rev | AACCCCGATTGAGATATAGATTTATTTAAATGGTTATGATAGTTATTGCTCAGCG |
| pACEBac1.rev | GGATCCGCGCCCGATGGTG |
| pACEbac1.for | AAGCTTGTCGAGAAGTACTAGAGGATCATAATCAGC |
| EcB_acebac1.rev | CTAGTACTTCTCGACAAGCTTTTATCGATTGTTTTGCCCCGCAG |
| EcB_acebac1.for | CATCGGGCGCGGATCCATGGGTAAACTAACGCTGCTGTTG |
| EcL_pAcebac.for | CATCGGGCGCGGATCCATGATCAGCAGAGTGACAGAAGCTC |
| EcL_pAcebac.rev | GTACTTCTCGACAAGCTTTTATTTTTGCACTACGATATTTTCTTGTGACGG |
| Ltripple.for | ATTAACAGAAGCTCTAAGCAAAG |
| Ltripple.rev | TTATTGATCATATGGGACTGAAAATAC |
| PaFtsL.fwd | ATTAGTTAAGTATAAGAAGGAGATATACATATGAGCCGTCTCTTCGTCAAGCG |
| PaFtsL.rev | CGCAGCAGCGGTTTCTTTTCATGGCGCCACCATCCTGAC |
| pET-Duet_FtsL.rev | TATGTATATCTCCTTCTTATACTTAACTAATATACTAAGATGGGGAA |
| pET-DuetFtsLfwd | AAAGAAACCGCTGCTGCGA |
| PaFtsQB.fwd | TTTTGTTTAACTTTAAGAAGGAGATATACCATGCATCACCATCACCACCACG |
| PaFtsQB.rev | ACTTTCTGTTCGACTTAAGCATTTAGTGGTGGTGGTGGTGATGGCT |
| pET-Duet_FtsQBfwd | ATGCTTAAGTCGAACAGAAAGTAATCGTATTG |
| pET-Duet_FtsQB.rev | GGTATATCTCCTTCTTAAAGTTAAACAAAATTATTTCTAGAGGG |
| SUMO-PaFtsW.fwd | TTTTGTTTAACTTTAATAAGGAGATATACCATGCATCATCACCACCACCACG |
| SUMO-PaFtsW.rev | AATTCAGTTTCATATGTATATCTCCTTCTTATACTTAACTAATATACTAAGATGGGGAAT |
| PaFtsI.fwd | AGTTAAGTATAAGAAGGAGATATACATATGAAACTGAATTATTTCCAGGGCGCC |
| PaFtsI.rev | GCAGCAGCCTAGGTTAATTATCAGCCACGCCCTCCTTTTGCG |
| pET-Colafwd | TAATTAACCTAGGCTGCTGCCACC |
| pET-Cola.rev | GGTATATCTCCTTATTAAAGTTAAACAAAATTATTTCTACAGGGG |
| Strep_PaFtsWI.rev | ACTACCTGCGCTACCTTTTTCAAACTGCGGATGTGACCATGCTGTCATGCCACCAATCTGCTCTCTATG |
| Strep_PaFtsWI.fwd | GCAGCAGGTAGCGGTGCAGGTTGGAGCCATCCTCAGTTTGAGAAAGGCCTGGAAGTGTTGTTC |
| PaFtsW_D275A.fwd | CCAGAGGCGCATACCGCTTTTGTGTTTGCGGTTC |
| PaFtsW_D275A.rev | GAACCGCAAACACAAAAGCGGTATGCGCCTCTGG |

**Supplementary Table S3: Plasmids**

| **Plasmid** | **Description** | **Reference** |
| --- | --- | --- |
| pLK1 | pET-Duet expression vector containing His_6_-SUMO-*Pa*FtsQ, *Pa*FtsB-His_6_ and *Pa*FtsL, AmpR | This study, based on^7^ |
| pLK2 | pET-Cola expression vector containing His_6_-SUMO-FLAG-3c-*Pa*FtsW and *Pa*FtsI, KanR | This study, based on^7^ |
| pLK3 | expression vector containing His_6_-*Sc*Ulp1^403-612^, CmrR | This study, based on^7^ |
| pLK4 | pET-Cola expression vector containing His_6_-SUMO-Strep-3c-*Pa*FtsW and *Pa*FtsI, KanR | This study |
| pLK9 | pLK4 with FtsW^D275A^ mutation | This study |
| pFE658 | *Ec*FtsB in pACEBac1, GenR | This study |
| pFE661 | Addition of gCATATG at 5’ end of BamHI in pLIB, GenR/AmpR | This study |
| pFE668 | *Ec*FtsI in pFE661, GenR/AmpR | This study |
| pFE674 | HisTEV_*Ec*FtsL in pLib, GenR/AmpR | This study |
| pFE686 | *Ec*FtsQ in pFE661, GenR/AmpR | This study |
| pFE749 | *Ec*FtsQ + *Ec*FtsB + His-TEV-*Ec*FtsL in pBig1a, SpecR/GenR/AmpR | This study |
| pFE756 | TwinStrep-*Ec*FtsW-FtsI in pBig1b, SpecR/GenR/AmpR | This study |
| pFE758 | TwinStrep-*Ec*FtsW-FtsI + *Ec*FtsQ + *Ec*FtsB + His-TEV-*Ec*FtsL in pBig2ab, CmrR/GenR/AmpR | This study |
| pNJ069 | Strep-TEV-*Ec*FtsK^1-222^, *Ec*FtsQ-His_6_ in pBig1a, AmpR/SpecR/GentaR | This study |

**Supplementary Table S4: Species with linked WI**

| **Species** |
| --- |
| *Candidatus* |
| *Clostridia bacterium* |
| *Actinobacteria bacterium 66 15* |
| *Coriobacteriaceae bacterium EMTCatB1* |
| *Coriobacteriia bacterium* |
| *Slackia sp. CM382* |
| *Slackia heliotrinireducens* |
| *Slackia isoflavoniconvertens* |
| *Parvibacter caecicola* |
| *Enterorhabdus mucosicola* |
| *Enterorhabdus caecimuris* |
| *Adlercreutzia equolifaciens* |
| *Denitrobacterium detoxificans* |
| *Eggerthellaceae bacterium AT8* |
| *Cryptobacterium curtum* |
| *Eggerthella sp. CAG 368* |
| *Eggerthella sp. CAG:209* |
| *Eggerthella sp. CAG:1427* |
| *Cryptobacterium sp. CAG:338* |
| *Gordonibacter sp. Marseille-P4307* |
| *Adlercreutzia sp. Marseille-P7992* |
| *Coriobacteriaceae bacterium 68-1-3* |
| *Raoultibacter timonensis* |
| *Senegalimassilia sp. KGMB04484* |
| *Senegalimassilia anaerobia* |
| *Bacteroides caecimuris* |
| *Gordonibacter urolithinfaciens* |
| *Enteroscipio rubneri* |
| *Paraeggerthella hongkongensis* |
| *Eggerthella timonensis* |
| *Coriobacteriaceae bacterium CHKC1002* |
| *Rubneribacter badeniensis* |
| *Bacteroides* |
| *Catonella morbi* |
| *Lachnoclostridium* |
| *Lachnospiraceae bacterium* |
| *Roseburia sp. CAG:100* |
| *Peptostreptococcaceae bacterium oral taxon 113 str. W5053* |
| *Rhodococcus* |
